# Mechanical regulation of epigenetics in vascular biology and pathobiology

**DOI:** 10.1111/jcmm.12031

**Published:** 2013-04-03

**Authors:** Li-Jing Chen, Shu-Yi Wei, Jeng-Jiann Chiu, C-M Cheng, M-J Tang

**Affiliations:** a Institute of Cellular and System Medicine, National Health Research InstitutesMiaoli, Taiwan; b Institute of Molecular Medicine, National Tsing Hua UniversityHsinchu, Taiwan

**Keywords:** Epigenetics, Mechanotransduction, Shear stress, Endothelial cells, Smooth muscle cells

## Abstract

Vascular endothelial cells (ECs) and smooth muscle cells (VSMCs) are constantly exposed to haemodynamic forces, including blood flow-induced fluid shear stress and cyclic stretch from blood pressure. These forces modulate vascular cell gene expression and function and, therefore, influence vascular physiology and pathophysiology in health and disease. Epigenetics, including DNA methylation, histone modification/chromatin remodelling and RNA-based machinery, refers to the study of heritable changes in gene expression that occur without changes in the DNA sequence. The role of haemodynamic force-induced epigenetic modifications in the regulation of vascular gene expression and function has recently been elucidated. This review provides an introduction to the epigenetic concepts that relate to vascular physiology and pathophysiology. Through the studies of gene expression, cell proliferation, angiogenesis, migration and pathophysiological states, we present a conceptual framework for understanding how mechanical force-induced epigenetic modifications work to control vascular gene expression and function and, hence, the development of vascular disorders. This research contributes to our knowledge of how the mechanical environment impacts the chromatin state of ECs and VSMCs and the consequent cellular behaviours.

- Introduction
- Vascular mechanobiology
  – Shear stress
  – Shear stress regulates physiological functions
  – Tensile force
- Epigenetics
  – Methylation
  – Histone modification and chromatin remodelling
  – RNA-based mechanisms
- Haemodynamic force-induced epigenetic modifications
  – Methylation
  – Histone acetylation/deacetylation (HAT/HDAC)
  – MicroRNA
- Conclusions and future perspectives

## Introduction

The human body is constantly exposed to various types of mechanical forces, such as the stretching of skeletal muscle, the compression of cartilage and bone and the haemodynamic forces on blood vessels 1. Haemodynamic forces are generated from the pulsatile nature of normal blood pressure and blood flow which can be characterized as cyclic stretch, shear stress and hydrostatic pressure 2. Although vascular endothelial cells (ECs) and vascular smooth muscle cells (VSMCs) are exposed to both cyclic stretch and shear stress, ECs are primarily subjected to shear stress resulting from blood flow, whereas VSMCs are subjected to cyclic stretch resulting from pulsatile blood pressure. These haemodynamic forces are sensed by mechanoreceptors, which play an initial role in sensing various mechanical stimuli as signals that are then transmitted to the interior of the cell *via* intracellular signalling pathways. This process is known as mechanotransduction 3. Many putative mechanoreceptors have been proposed, including ion channels, integrins, receptors of tyrosine kinases (RTKs), G protein coupled receptors, apical glycocalyx, primary cilia and adhesion molecules. In response to various mechanical stimuli, these mechanoreceptors signal through adaptor molecules to activate upstream signalling molecules, such as Ras, which then mediate intracellular signalling through phosphorylation cascades, eventually leading to the morphological and functional changes to maintain homeostasis. These changes include the regulation of gene expression, differentiation, proliferation, angiogenesis and migration. Vascular cell dysfunction because of the impairment of these changes may lead to a pathophysiological state that contributes to the development of vascular disorders, such as atherosclerosis and hypertension 4.

Since Conrad Waddington first proposed the concept of ‘epigenetics’ in 1942, research has advanced from genotype to phenotype 5. Epigenetics refers to the study of heritable changes in gene expression and phenotype (*i.e*. appearance) that occur without changes in the DNA sequence; such changes regulate the dynamics of gene expression 6. Epigenetics offers a new perspective on gene regulation that broadens the classic cis/trans paradigm of transcriptional processes and helps to explain unresolved problems from limitations of gene expression 6. Extensive evidence has revealed that epigenetic processes play crucial roles in the development of various diseases, including cancers and cardiovascular and neurological disorders 7. Studies investigating the role of epigenetics in vascular biology and pathophysiology have emerged only recently. The key processes that comprise epigenetic regulation are DNA methylation, histone modification/chromatin remodelling and post-transcriptional gene regulation by RNA-based mechanisms, such as non-coding RNAs (ncRNAs) 6. DNA methylation is the addition of a methyl group from S-adenyl methionine (SAM) to the fifth carbon of a cytosine residue to form 5-methylcytosine (5-mC) in the context of CpG dinucleotides 8. The hypermethylation of CpG islands results in the stable silencing of gene expression. Histone proteins are modified by lysine histone acetyltransferases (HATs) or histone deacetylases (HDACs) at their N-terminal regions, a process that influences the accessibility of the DNA to the transcriptional machinery 9. NcRNAs, such as microRNAs (miRNAs), are recently emerging endogenous, non-coding, single-stranded RNAs of 18–22 nucleotides that constitute a novel class of gene regulators. MiRNAs bind to their target genes within their 3′-untranslated regions (3′-UTRs), leading to the direct degradation of the messenger RNA (mRNA) or translational repression by a perfect or imperfect complement respectively 10.

Here, we discuss epigenetics as a complex interaction between the genome, surrounding environment and mechanical forces, such as haemodynamic forces, in vascular physiology and pathophysiology. This article gives an introduction and provides new insights into the role of mechanical force-induced epigenetic modifications in vascular cell gene expression, function and pathophysiology by presenting studies of eNOS gene expression, differentiation, angiogenesis, migration, atherosclerosis and hypertension. We also provide *in vivo* evidence that documents the importance of epigenetic modifications in EC and VSMC gene expression and function in response to haemodynamic force. In conclusion, we propose haemodynamic force to be a critical epigenetic manipulator in modulating vascular biology and pathophysiology in health and disease.

## Vascular mechanobiology

**Fig. 1:**
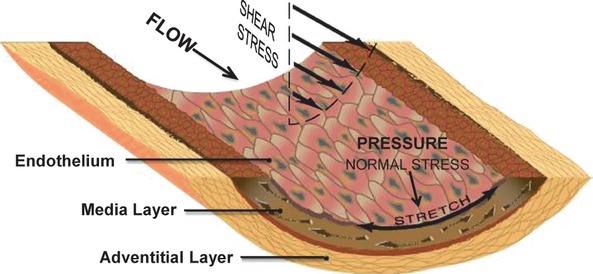
Schematic diagram showing the generations of shear stress (parallel to the endothelial surface), normal stress (*i.e*. pressure; perpendicular to the endothelial surface), and circumferential stretch because of blood flow and pressure (from Chiu and Chien 2).

Blood vessels are constantly exposed to various types of haemodynamic forces, including fluid shear stress, cyclic stretch and hydrostatic pressure, which are induced by the pulsatile nature of blood flow and pressure 2. Fluid shear stress is the frictional force per *unit* area from flowing blood and acts on the ECs present on the luminal surface of the vessel 11. Cyclic stretch arises because of blood pressure, causing circumferential stretching of the vessel wall and affects both the ECs and the VSMCs that surround the endothelium in arteries (Fig. 1) 2, 4. Hydrostatic pressure *per se* might also alter cellular physiology, but it is less important than shear stress or cyclic stretch. An increasing number of studies indicate that haemodynamic forces utilize mechanotransduction to influence endothelial physiology, the morphology of the embryonic heart and blood vessels and atherosclerosis 3. In this section, we discuss the cellular response to shear stress and tensile stress in ECs and VSMCs respectively.

### Shear stress

#### Shear stress modulates vascular morphogenesis

The pattern of blood flow in the development of heart tissue and vessels has been shown to play a critical role in vascular morphogenesis. By analysing intracardiac flow forces in zebrafish embryo, which is an ideal model for investigating the cellular and molecular events of cardio-vasculogenesis because of the ability to visually inspect the embryo and the accessibly of genetic modifications 12, Hove *et al*. demonstrated that the blood flow influences the development of the heart and vascular vessels 13. Furthermore, Lucitti *et al*. provided elegant evidence that fluid shear stress mediates the rearrangement of a primitive vascular plexus into a mature vascular tree in early mouse embryos 14. The latter study suggested that fluid shear stress-induced vessel remodelling is mediated by nitric oxide synthase 3 (NOS3, endothelial NOS, eNOS). North *et al*. 12 and Adamo *et al*. 13 demonstrated that fluid shear stress is able to promote embryonic haematopoiesis, increase the expression of haematopoietic markers and induce colony formation. Knockdown of eNOS decreased the ability of haematopoiesis in haematopoietic stem cells (HSCs). The finding that fluid shear stress-induced eNOS enhances haematopoiesis has been found to be conserved from fish to mammals 15, 16.

#### Shear stress regulates physiological functions

In addition to vascular morphogenesis and haematopoiesis, shear stress-induced mechanotransduction in ECs also regulates cellular functions, including cell proliferation/survival, metabolism, cytoskeletal reorganization and cell morphology 11. For *in vitro* studies, a parallel-plate flow channel is created using a gasket with a rectangular cut-out that is made with a thin silicon membrane and has a uniform channel height along the flow path 11. The parallel-plate flow channel can be used to study the effects of steady shear at 12 dyne/cm^2^, ‘static’ control with shear stress at 0.5 dyne/cm^2^, pulsatile shear at 12 ± 4 dyne/cm^2^, and reciprocating shear (oscillatory) at 0.5 ± 4 dyne/cm^2^. Disturbed shear is generated in a step-flow channel 17. Interestingly, different patterns of shear stress cause the opposite result with respect to the functions. In laminar shear (steady shear), many events are transiently induced, including the production of reactive oxygen species (ROS), activation of GTPases and pro-inflammatory pathways, such as JUN N-terminal kinase (JNK) and NF-κB 18, and production of adhesion molecules, such as monocyte chemotactic protein-1 (MCP-1) 19. These events eventually decrease to substantially below baseline levels compared with static controls. In contrast, these events are continuously stimulated by disturbed shear and oscillatory shear 4, 20. Cell cycle regulators, such as p53 and p21, are up-regulated by laminar shear, leading to cell cycle arrest 21. In disturbed shear and oscillatory shear, bromodeoxyuridine (BrdU) incorporation is markedly enhanced, resulting in increased cell proliferation 20. Once ECs are exposed to laminar shear, their cytoskeletal fibres undergo remodelling to align the cell in the direction of the shear flow. This remodelling of cytoskeletal fibres is not observed under disturbed flow, but the cells instead appear in a random orientation, similar to that observed under static conditions 17, 22. The cdc42 GTPase and the Rho signalling pathway are involved in shear stress-induced cytoskeletal remodelling 23, 24.

#### Shear stress is involved in the development of vascular pathologies

Endothelial dysfunction may lead to a pathophysiological state that contributes to the development of vascular disorders, including atherosclerosis and thrombosis and their complications 2, 4. The possible role of haemodynamic forces in endothelial dysfunction was first suggested on the basis of the observation that the earliest atherosclerotic lesions characteristically develop at arterial branches and curvatures where the shear is low and disturbed 2. These areas include the carotid bifurcations and the branch points of the coronary, infrarenal and femoral arteries. Recent studies indicate that disturbed shear and oscillatory shear induce sustained activation of a number of atherogenic genes in ECs to promote the development of atherosclerosis 2, 4. Disturbed shear induces EC dysfunction, resulting in the expression of adhesion molecules, such as intercellular adhesion molecule-1 (ICAM-1), vascular cell adhesion molecule-1 (VCAM-1) and E-selectin (E-sel) and chemokines, such as MCP-1. Together, these adhesion molecules and chemokines recruit leukocytes and monocytes, thereby initiating a pro-inflammatory process within the vessel wall. KLF-2 is a key shear stress-induced transcription factor that governs the expression of shear stress-induced genes in ECs 25. When ECs are subjected to laminar shear stress, KLF-2 is induced and plays anti-inflammatory and anticoagulant roles. In contrast, disturbed shear and oscillatory shear diminish the expression of KLF-2 and cause the dysfunction of ECs 26. Based on the above *in vitro* studies, disturbed/oscillatory shear and laminar shear induce differential molecular responses in ECs, leading to preferential sites of atherosclerotic lesion formation.

### Tensile force

Unlike the well-established responses of ECs subjected to fluid shear stress, the VSMCs response to cyclic stretch is less clear; however, the two processes share many similar features. As with shear stress, the mechanoreceptors, such as integrins, RTKs and ion channels, can sense tensile force from blood pressure and transmit the stimuli into intracellular signalling pathways 27. Several reports have described the important role of cyclic stretch on VSMC gene expression and cellular functions, such as proliferation/apoptosis, migration/alignment and differentiation (phenotypic switch) 28. In conditions of abnormal blood pressure, such as hypertension, the vascular wall is chronically subjected to exaggerated tensile force by high blood pressure, leading to vascular remodelling, arterial stiffness and calcification 29.

#### Cyclic stretch modulates gene expression in VSMCs

During arterial remodelling, the matrix metalloproteinases (MMPs) play a prominent role in mediating changes to the extracellular matrix (ECM). Asanuma *et al*. demonstrated that human cultured VSMCs subjected to physiological levels (5%) of stationary or cyclical (1 Hz) uniaxial cyclic stretch had significantly decreased protein and mRNA levels of MMP-2 and MMP-9 after 48 hrs 30. This report indicates that VSMCs selectively respond to different types of cyclic stretch and the cyclic stretch-induced alteration in MMPs may be involved in the remodelling of the ECM surrounding the vasculature.

#### Cyclic stretch regulates the functions of VSMCs

Vascular smooth muscle cells hypertrophy, hyperplasia, migration and ECM remodelling are considered to be a key in the development of hypertension. Watase *et al*. used a custom-designed plexiglass pressure chamber and subjected VSMCs to 105 or 120/90 mm Hg pressure at a frequency of 60 cycles/min. (0.5 sec. systole, 0.5 sec. diastole) 31. Interestingly, VSMCs displayed a more elongated morphology and a significant increase in cell number when they were continuously exposed to pressure until day 9. This is the first study to reveal the role of cyclic stretch in modulating the phenotypic modification of VSMCs. Furthermore, Birukov *et al*. demonstrated that cyclic stretch (30 cycles/min.; 15% elongation) induced the proliferation of VSMCs and increased the expression of the specific contractile protein h-caldesmon in VSMCs 32. These data suggest that cyclic mechanical stimulation has dual effects on VSMCs to modulate their proliferation and differentiation. Moreover, uniaxial cyclic stretch causes VSMCs to align in a direction that is perpendicular to the direction of cyclic stretch. The mechanism of cyclic stretch-induced cytoskeletal remodelling and alignment remains to be elucidated 28. Li *et al*. further demonstrated that cyclic stretch (60 cycles/min.; 5, 15, or 20% elongation) enhances VSMC migration by promoting the translocation of protein kinase C-δ (PKC-δ) to the cytoskeleton 33. PKC-δ-deficient VSMCs, which were cultured from PKC-δ^−/−^ mice, were unable to migrate in response to cyclic stretch. This study indicates that PKC-δ is a key signal transducer in the modulation of VSMC migration.

## Epigenetics

The original concept of epigenetics was coined by Conrad H. Waddington. In 1939, in his student handbook entitled ‘An Introduction to Modern Genetics’, he suggested ‘the causal interactions between genes and their products, which bring the phenotype into being’ 5. More recently, epigenetics has been redefined as the study of heritable changes in gene expression or cellular phenotype that occur without alterations in the DNA sequence 6. These changes are achieved by covalent and non-covalent modifications of DNA and histone proteins-mediated modifications of the entire chromatin structure. Epigenetic modifications can be classified into the following three main categories: DNA methylation, histone modification/chromatin remodelling, and RNA-based mechanisms. RNA-based mechanisms are a newly recognized type of epigenetic modification in which gene expression is regulated by ncRNAs.

### Methylation

DNA methylation was first discovered in mammals and occurs *via* the addition of a methyl group from SAM to the fifth carbon of a cytosine residue to form 5-mC 8. DNA methylation occurs almost exclusively in the context of CpG dinucleotides. CpG dinucleotides tend to cluster into so-called CpG islands 34, which are defined as a region of greater than 200 bases with a CG content of at least 50% and a ratio of statistically expected CpG frequencies of at least 0.6. CpG dinucleotides are quite rare in mammalian genomes (∼1%). In the human genome, ∼60% of gene promoters are associated with CpG islands, and in normal cells, these islands are generally unmethylated 7. The methylation of CpG islands results in the stable silencing of gene expression. During early embryonic development, CpG islands undergo differential methylation 35. These marks are important for early embryonic development and the establishment of totipotency or pluripotency as well as for health later in life. The methylation of CpG islands plays a crucial role in genomic imprinting. The mechanisms of DNA methylation include the following three steps: enzymes catalyse the addition of a methyl group onto cytosine (methylation), enzymes remove the methyl group (demethylation), and methylation-associated proteins recognize and bind to the methyl group to eventually influence gene expression 8. DNA methylation is catalysed by a family of DNA methyltransferases (DNMTs), which includes DNMT1, DNMT2, DNMT3a, DNMT3b and DNMT3L; however, only DNMT1, DNMT3a and DNMT3b possess methyltransferase activity 7. DNMT-3L lacks intrinsic methyltransferase activity, but it is able to interact with DNMT3a and 3b, leading to the methylation of retrotransposons 36. DNA methylation-associated proteins, including methyl CpG-binding domain (MBD) proteins, ubiquitin-like PHD and RING finger domain (UHRF)-containing proteins, and zinc-finger domain proteins, can bind to 5-mC with high affinity to modulate gene transcription *via cis* and *trans* interactions. In vascular ECs, the methylation of CpG islands in promoters of eNOS and VEGFR2 (vascular endothelial growth factor receptor 2) are identified and MBD2 can bind to these methylated CpG islands and suppresses the gene expression. Loss of MBD2 leads to activation of eNOS and VEGFR2 gene expression, and trigger proangiogenetic signal pathway 37. This evidence provides the importance of DNA methylation in vascular function.

### Histone modification and chromatin remodelling

In the nuclei of eukaryotic cells, genomic DNA is packaged into chromatin, which is composed of DNA and proteins. The unit of chromatin is the nucleosome, which consists of an octamer of four core histone proteins (H2A, H2B, H3 and H4) that are wrapped around ∼147 base pairs of DNA in 1.64 left-handed turns 9. There are 14 contact points between the histones and DNA per nucleosome 38. The striking feature of histones is their N-terminal ‘tails’, which are unstructured. A large number and different types of modified residues are placed on the tail of the histone. All of the histones are subjected to post-transcriptional modifications. There are at least eight distinct types of modifications, including lysine acetylation, lysine and arginine methylation, serine and threonine phosphorylation, lysine ubiquitylation, lysine sumoylation, ADP ribosylation, arginine deimination and proline isomerization 9. In general, acetylation, methylation, phosphorylation, and ubiquitylation of histones have been implicated in the activation of transcription; whereas methylation, ubiquitination, sumoylation, deimination, and proline isomerization of histones have been implicated in the repression of transcription. Among these modifications, histone acetylation is most well studied. In mammalian cells, acetylation is directly performed by histone acetyltransferases (HATs) from acetyl-coenzyme A complexes. HATs are divided into three families, including GNAT, MYST and CBP/p300 9, 39. Histone tails are acetylated by HATs, resulting in the neutralization of their positive charge and the relaxation of the chromatin structure. This change in the chromatin structure increases the accessibility of transcription factors to their target genes. Spin *et al*. further demonstrated that p300 is involved in regulation of VSMC phenotypic switch and implicates the complex role of p300 in chromatin remodelling 40. In contrast, acetyl groups can be removed from histones by histone deacetylases (HDACs). There are three distinct families of HDACs: class I (HDAC1-3 and HDAC8), class II (HDAC4-7 and HDAC9-10) and class III [NAD-dependent enzymes of the sirtuin (SIRT) family (SIRT1-7)] 41. Class I HDACs are expressed ubiquitously in the nucleus and display high enzymatic activity. Class II HDACs are further subdivided into IIA and IIB. Class IIA HDACs (HDAC4-5, HDAC7 and HDAC9) have long N-terminal extensions with conserved binding sites for the transcription factor myocyte enhancer factor-2 (MEF-2) and the chaperone protein 14-3-3, which regulates nuclear-cytoplasmic shuttling 42. Class IIA HDACs can be phosphorylated by kinases, thereby providing a mechanism for linking extracellular signals with transcription. Class IIB HDAC6 is the primary cytoplasmic deacetylase found in mammalian cells, whereas the functions of HDAC10 are less known. Class III HDACs represent the silent information regulator 2 (Sir2) family of nicotinamide adenine dinucleotide (NAD+)-dependent HDACs (*i.e*. SIRT1-7), which share structural and functional similarities with yeast Sir2 43. Histone acetylation is a dynamic process that is controlled by the antagonistic actions of two large families of enzymes 41. The balance between these actions represents a critical regulatory mechanism for gene expression, developmental processes and disease progression. Recently, HDACs and HDAC inhibitors have been suggested as clinical therapies for several diseases, such as cancer, cardiovascular diseases, Huntington's disease and Alzheimer's disease 43–45. HDAC inhibitors will likely widen the therapeutic window and possibly lead to their clinical application 46.

In addition to histone modifications, chromatin remodelling can be achieved by ATP-dependent chromatin remodelling complexes 47. These chromatin remodelling complexes utilize ATP hydrolysis to alter the histone-DNA interaction. The consequences of chromatin remodelling lead to the transient unwrapping of the DNA from the histones, the formation of a DNA loop and the removal of the nucleosome and histone variants; each of these processes results in changes in the accessibility of nucleosomal DNA to transcription factors. These alterations in chromatin structure lead to changes in transcription in a wide variety of biological processes and provide a complex and responsive epigenetic landscape that is superimposed on the underlying genetic code.

### RNA-based mechanisms

The newly recognized type of epigenetic modification for gene regulation involves ncRNA. NcRNA is functional RNA that is not translated into protein. The functionality of individual ncRNAs has been found in mammals, other animals, plants and fungi. Recent studies reveal that ncRNAs are involved in the regulation of various processes, such as metabolism, development, cell proliferation and oncogene induction 48. The following five classes of non-coding RNAs have been defined: microRNAs (miRNAs), small interfering RNAs (siRNAs), piwi-interacting RNAs, small nucleolar RNAs and long non-coding RNAs (lncRNAs) 48, 49. The functions and features of lncRNAs are distinct from other small ncRNAs, such as miRNAs. Xist nuclear RNA is a 17 kb lncRNA, which is expressed exclusively from the inactive X chromosome in women 50. MiRNAs are recently emerging endogenous, non-coding, single-stranded RNAs of 18–22 nucleotides that constitute a novel class of gene regulators. MiRNAs bind to their target genes within their 3′-untranslated regions (3′-UTRs), leading to the direct degradation of the mRNA or translational repression by a perfect or imperfect complement respectively 10. In recent years, the role of miRNAs has received increasing attention in the development of various diseases. Particularly, the functions of miRNAs in vascular development and diseases have been described 51, 52. Most miRNA genes are located in intronic regions, and they may be transcribed as part of the mRNA. The primary miRNA (pri-miRNA) is transcribed by either RNA polymerase II or III from an independent gene in the nucleus. In subsequent processing, the microprocessor complex (*i.e*. Drosha-DGCR8) processes the pri-miRNA into a ∼60–100-nucleotide precursor hairpin (pre-miRNA) 53. The resulting pre-miRNA is exported to the cytoplasm by Exportin-5-RanGTP. In the cytoplasm, the RNase III Dicer and TRBP cleave the pre-miRNA into ∼22-nucleotide miRNA/miRNA* duplexes 54. The miRNA strand is termed the guide strand and represents the mature miRNA; the miRNA* strand is termed the passenger strand and undergoes rapid degradation 55. The mature miRNA is incorporated into a miRNA-induced silencing complex and base-paired to its target mRNA for mRNA degradation or translational repression. Several miRNAs have been identified to play important role in vascular vessel. The dual functions of miR-126, which is the most abundant miRNA in vascular ECs, have been demonstrated in angiogenesis and anti-inflammation. During the embryonic development, miR-126 has been shown to regulate the angiogenic signalling and to govern the integrity of blood vessel 56. Overexpression of miR-126 significantly represses TNF-α-induced VCAM-1 expression and leucocyte adhesion 57. MiR-143/145 are specifically expressed in normal blood vessel and involved in the modulation of VSMC fate in differential phenotype 58.

## Haemodynamic force-induced epigenetic modifications

Although extensive studies have demonstrated that haemodynamic forces modulate various vascular cell functions, reports of haemodynamic force-induced epigenetic regulation of gene expression, function and vascular pathophysiology have recently emerged. In this section, we will discuss these mechanisms and functions of haemodynamic force-induced methylation, histone modifications and microRNAs in vascular cells (Tables 1 and 2).

**Table 1:** HDACs involved in the regulation of vascular cells in response to haemodynamic forces

DHACs	Co-factor	Type of mechanical factor	Cell type	Functions	References
HDAC1	p53	Laminar flow(12 dyne/cm^2^)	ECs	Deacetylation of p53 and activation of p21	65
	Nrf-2	Oscillatory flow (0.5 ± 4 dyne/cm^2^)	ECs	Promotion of proliferation and oxidation	66
HDAC2	Nfr-2	Oscillatory flow (0.5 ± 4 dyne/cm^2^)	ECs	Promotion of proliferation and oxidation	66
HDAC3		Laminar flow(12 dyne/cm^2^)	EPCs	EPCs differentiate into ECs	68
	Akt	Disturbed flow (mean 4.5 dyne/cm^2^)		Cell survival and knockout of HDAC3 leads to formation of atherosclerosis	67
	Nrf-2/MEF-2	Oscillatory flow (0.5 ± 4 dyne/cm^2^)	ECs	Promotion of proliferation and oxidation	66
HDAC4		Laminar flow(10 dyne/cm^2^)	ECs	Mediation of flow-induced NO production	71
HDAC5		Laminar flow(10 dyne/cm^2^)	ECs	Mediation of flow-induced NO production	71
	MEF-2	Laminar flow(24 dyne/cm^2^)	ECs	Phosphorylation of HDAC5, enhances MEF-2 transcriptional activity, leading to eNOS production	72
	MEF-2	Oscillatory flow (0.5 ± 4 dyne/cm^2^)	ECs	Cell proliferation	66
HDAC6		Laminar flow(15 dyne/cm^2^)	ECs	Low level of acetylated tubulin, regulates migration of VSMCs	73
HDAC7	MEF-2	Oscillatory flow (0.5 ± 4 dyne/cm^2^)	ECs	Cell proliferation	66
		Cyclic strain of 1 Hz at 10%	VSMCs	Inhibition of migration	74
SIRT1		Laminar flow(12 dyne/cm^2^)	ECs	Enhance eNOS production	75

**Table 2:** MiRNAs involved in the regulation of vascular cells in response to haemodynamic forces

miRNA	Expression	Type of mechanical factor	Cell type	Functions	References
Let-7d	Decreased	SHR rats	VSMCs	Anti-proliferation	91
miR-10a	Decreased	*In vivo* athero-susceptible regions	ECs	Anti-inflammation	85
miR-126	Increased	*In vivo* blood flow	ECs	Angiogenesis	82
miR-130a	Increased	SHR rats	VSMCs	Proliferation	90
miR-19a	Increased	Laminar flow(12 dyne/cm^2^)	ECs	Anti-proliferation	83
miR-21	Increased	Oscillatory flow (0.5 ± 4 dyne/cm^2^)	ECs	Pro-inflammation	87
miR-23b	Increased	Pulsatile flow (12 ± 0.4 dyne/cm^2^)	ECs	Anti-proliferation	84
miR-26a	Increased	Cyclic strain of 1 Hz at 12%	VSMCs	Hypertrophy	89
miR-663	Increased	Oscillatory flow (0.5 ± 4 dyne/cm^2^)	ECs	Pro-inflammation	86
miR-92a	Increased	Oscillatory flow (0 ± 4 dyne/cm^2^)	ECs	Atherogenesis	81

### Methylation

Constitutive eNOS expression in ECs is dependent on the basal transcriptional machinery present in its core promoter and includes positive and negative protein-protein (*trans*) and protein-DNA (*cis*) interactions 59. The haemodynamic force-induced regulation of eNOS gene expression in mRNA processing and stability has been clarified. Recent studies indicate that shear stress can modulate chromatin remodelling on histone H3 and H4, resulting in eNOS being regulated by chromatin-based epigenetic mechanisms at the transcriptional level 60, 61. Lund *et al*. found that DNA hypermethylation patterns occur prior to the appearance in peripheral blood mononuclear cells, human monocyte cell line THP-1, and the aorta of ApoE mice 62. The latter study was the first analysis of DNA methylation at the early stages of atherosclerosis and the first description of genomic DNA sequences undergoing epigenetic changes in a mouse model. Kim *et al*. further demonstrated the DNA methylation profiles from atherosclerotic tissue and the DNA methylation patterns of estrogen receptor-β, which has been identified to have an important role in atherosclerotic development 63. Atherosclerotic tissues showed higher methylation levels (28.7%) than normal arteries (6.7–10.1%) and venous tissues (18.2%), and the methylation of estrogen receptor-β could be diminished with a DNA methyltransferase inhibitor 64.

### Histone acetylation/deacetylation (HAT/HDAC)

#### Class I HDACs

**Fig. 2:**
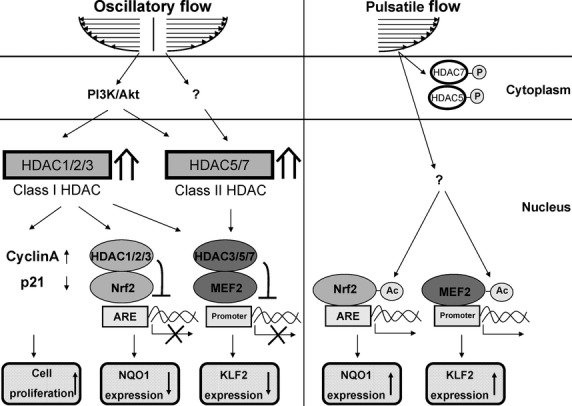
Schematic diagram of HDAC signalling and its modulation of gene expression and function in ECs in response to oscillatory and pulsatile flow. Oscillatory flow induces the expression and nuclear accumulation of class I HDAC1/2/3 and class II HDAC5/7 in ECs. Oscillatory flow induces associations of HDAC1/2/3 with Nrf-2, causing the deacetylation of Nrf-2 and inhibition of binding of Nrf-2 to the antioxidant response element (ARE), which result in the repression of NQO1 expression. Furthermore, HDAC1/2/3 are involved in the oscillatory flow-induced cell cycle progression and proliferation of ECs. In addition, oscillatory flow induces associations of HDAC3/5/7 with MEF-2, which lead to the inhibition of KLF-2 expression as a result of MEF-2 deacetylation. In contrast, pulsatile flow induces the phosphorylation-dependent nuclear export of HDAC5/7 in ECs, and hence induces the expressions of NQO-1 and KLF-2 (from Lee *et al*. 65).

Haemodynamic force-induced histone modifications have been extensively studied in recent years (Table 1). These histone modifications are involved in the regulation of gene expression, cellular functions, including cell proliferation, survival, migration and atherosclerosis. Zeng *et al*. demonstrated that laminar flow increases the activity of HDACs and the association of p53 with HDAC1, leading to the deacetylation of p53 in ECs 65. Treating ECs with Trichostatin A (TSA), an HDAC inhibitor, abolishes the flow-induced p53 deacetylation at Lys-320 and Lys-373. Furthermore, HDAC-deacetylated p53 by laminar shear stress triggers the expression of p21, whereas deletion and mutation of the p21 promoter inhibits p53 activation. These data clearly outline the mechanisms of laminar shear stress-induced cell cycle arrest. Lee *et al*. utilized HDAC-specific siRNAs to find that class I HDAC1/2/3, but not class II HDAC4/7, modulate oscillatory flow-induced cell proliferation 66. Oscillatory flow up-regulates the expression of cyclin A and down-regulates the expression of p21 through class I HDAC1/2/3, resulting in the promotion of EC proliferation. In an *in vivo* stenosis model in which the rat abdominal aorta was subjected to partial constriction with a U-clip, which produced a 65% constriction in diameter 26, high expression of HDAC2/3/5 and BrdU uptake were observed in the luminal ECs at post-stenotic sites, where disturbed flow with oscillatory shear occurs. In addition, when the HDAC inhibitor valproic acid (VPA) was injected into the rats infused with BrdU, the increased BrdU uptake in the ECs at the post-stenotic region was inhibited when compared with the group injected with saline. These results indicate that oscillatory flow-induced EC proliferation is mediated by class I HDACs *in vivo* (Fig. 2). Zampetaki *et al*. utilized *en face* staining to demonstrate increased levels of HDAC3 in aortas from apolipoprotein E (apoE)-knockout mice 67. In addition, cultured ECs were found to up-regulate the expression of HDAC3 proteins and to enhance its phosphorylation at serine/threonine residues in response to disturbed flow. Co-immunoprecipitation studies revealed that HDAC3 and Akt form a complex to promote EC survival. Knockdown the expression of HDAC3 *via* its specific short hairpin RNA (shHDAC3) led to a dramatic decrease in cell survival accompanied by EC apoptosis. These results indicate that disturbed flow promotes the post-transcriptional modification and stabilization of the HDAC3 protein, thereby highlighting its contribution to atherogenic processes. Zeng *et al*. demonstrated that laminar shear stress enhances embryonic stem cell-derived progenitor cell differentiation into ECs. This process stabilizes and activates HDAC3 through the Flk-1-PI3K-Akt pathway and deacetylates p53, resulting in p21 activation 68.

#### Class II HDACs

Chen *et al*. demonstrated that p300, which is a histone acetyltransferase, cooperates with NF-κB subunits (p50 and p65) to bind to the shear stress responsive B element in the human eNOS promoter 69. The shear stress-induced eNOS expression was blocked by pharmacological inhibition of p300/HAT activity with curcumin or p300-specific siRNA. Chromatin immunoprecipitation assays also revealed that shear stress stimulates the acetylation of histones H3 and H4 at the eNOS promoter, corroborating the results of a previous study 70. On the other hand, histone deacetylations have been demonstrated to play a critical role in shear stress-mediated eNOS expression. Application of laminar flow to ECs induces their production of NO, which promotes the deacetylation of histones, leading to the enhancement of class II HDAC4/5 nuclear shuttling and the increased activity in ECs 71. Wang *et al*. found that the phosphorylation of class II HDAC5 and its nuclear export were stimulated by laminar shear stress through a calcium/calmodulin-dependent pathway 72. Consequently, flow induced the dissociation of HDAC5 from MEF-2 and enhanced MEF-2 transcriptional activity, leading to KLF-2 and eNOS expression. In addition to regulation of eNOS expression by class II HDACs, Wang *et al*. used ECs co-cultured with VSMCs to demonstrate that HDAC6 is involved in the modulation of laminar shear stress-induced migration in ECs 73. The acetylation level of tubulin, which is an important cytoskeletal protein involved in the regulation of cell migration was decreased in these co-cultured ECs by shear stress. Yan *et al*. found that a cyclic stretch of 1 Hz at 10% elongation significantly inhibited the migration of cultured VSMCs, and this treatment up-regulated the levels of hyperacetylated histone H3 and HDAC7 and down-regulated the levels of HDAC3/4 74.

#### Class III HDACs

Chen *et al*. demonstrated the interplay of class III NAD-dependent enzymes, SIRT1 and AMP-activated protein kinase (AMPK) in the regulation of eNOS expression. Laminar shear stress and pulsatile flow increase the SIRT1-eNOS association and eNOS deacetylation. In addition, shear stress activates AMPK activity; hence, the phosphorylation of eNOS by AMPK is required for the SIRT1 deacetylation of eNOS, leading to the expression of eNOS 75. The class III HDAC SIRT1 has been also shown to play a protective role in atherosclerosis 76. SIRT1 deacetylates RelA/p65 at lysine 310 in macrophages and suppresses its binding to naked DNA in human aortic ECs, thereby interfering with a crucial step in NF-κB signalling and reducing the expression of EC adhesion molecules, including ICAM-1 and VCAM-1. Overexpression of endothelial SIRT1 in apoE-deficient mice prevents the formation of atherosclerosis by improving vascular function. In addition, SIRT1 is also involved in the proliferation and migration of VSMCs. The increased activity of SIRT1 in VSMCs leads to the suppression of p21 and enhancement of senescence-resistant cells replication 76. In addition, the activity of the tissue inhibitor of metalloproteinase-3 can be increased by SIRT1 overexpression. Therefore, SIRT1 plays a protective role in atherosclerosis in VSMCs by inhibiting the inflammatory events and thereby preventing atherosclerotic plaques formation.

Several HDAC inhibitors are studied in the spontaneously hypertensive rat (SHR) model. Cardinale *et al*. demonstrated that SHRs treated with VPA for 20 weeks had significant decreases in blood pressure, the levels of pro-inflammatory cytokines and hypertrophic markers, such as reactive oxygen species, and the expression of angiotensin II type 1 receptor in the heart. 77. Bogaard *et al*. also found that VPA and TSA reduce pressure overload-induced left ventricular hypertrophy and dysfunction, but the mechanisms of the effect on right ventricular adaptation to pressure overload are unknown 78. Usui *et al*. analysed the expression of proteins in the aorta and mesenteric artery from SHRs and Wistar Kyoto rats (WKYs) by Western blotting 79. The expression of HDAC4 and HDAC5 were decreased in SHRs compared with WKYs. In the mesenteric arteries from SHRs, HDAC4 was increased, whereas HDAC5 was decreased. Taken together, HDACs play an important role in the development of hypertension.

### MicroRNA

**Fig. 3:**
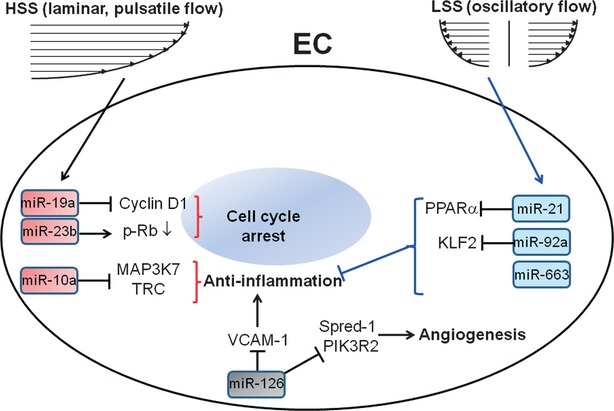
Schematic diagram of miRNAs and their function in ECs in response to shear stress. Oscillatory flow induces the expression of miR-92a, miR-21 and miR-663, leading to the repression of KLF-2 and PPAR-α expressions and an inflammatory response in ECs. Laminar shear stress induces the expressions of miR-19a and miR-23b, resulting in cell cycle arrest in ECs.

In current studies, the functions of haemodynamic force-induced miRNAs have been clarified in vascular cells (Fig. 3). These functions include angiogenesis, inflammation, proliferation and migration. The endothelium-specific transcription factor KLF-2 has been well-established to participate in the regulation of eNOS gene expression 80. A new regulatory circuit of KLF-2-mediated expression of eNOS by an RNA-based mechanism has been clarified 81. ECs that were subjected to oscillatory flow (0 ± 4 dyne/cm^2^), but not pulsatile flow (12 ± 4 dyne/cm^2^) were triggered to express miR-92a. Bioinformatics analysis demonstrated that KLF-2 is a target gene of miR-92a, and its gene and protein expression levels are down-regulated in oscillatory shear-stimulated ECs. In addition, the KLF-2-regulated genes eNOS and thrombomodulin were repressed by the overexpression of miR-92a in ECs. Nicoli *et al*. used a zebrafish embryonic model to demonstrate that the angiogenic sprouting of blood vessels requires the blood flow-induced transcription factor KLF-2 82. KLF-2 acts upstream of miR-126 to promote fluid flow-stimulated angiogenesis through VEGF signalling. Co-injection of both morpholinos, resulting in the specific knockdown of KLF-2 and miR-126, caused a dramatic defect in the penetrance of AA5X. This implies that KLF-2 and miR-126 share a common pathway in modulating angiogenesis. This study provided new insights into how ECs respond to flow stress and integrate developmental signals with miR-126 to promote angiogenesis. On the other hand, laminar shear stress has been identified as a regulator of EC anti-proliferation as a result of miRNA modulation of cell cycle regulators. Qin and Wang *et al*. demonstrated that laminar shear stress induces miR-19a and miR-23b, which may participate in cell cycle regulation, leading to EC arrest at G_1_/S 83, 84.

Fang *et al*. showed that swine vessels exhibit decreased expression of miR-10a at athero-susceptible regions of the inner aortic arch and aorta-renal branches, which are the preferential sites for atherosclerotic occurrence 85. This group further demonstrated that miR-10a is involved in the anti-inflammatory effect observed in ECs. As unusual fluid shear forces, such as disturbed flow and oscillatory flow, promote the development of atherosclerosis, the link between fluid shear stress and the development of atherosclerosis may involve regulation by miR-10. The detailed mechanisms of this regulatory circuit should be further explored. Ni *et al*. described the miRNA expression profiles of cultured ECs exposed to oscillatory flow and laminar flow 86. The overexpression of miR-663 increases monocyte adhesion in laminar flow-exposed ECs, whereas the treatment of ECs with a miR-663 antagonist inhibits oscillatory flow-induced monocyte adhesion. MiR-663 was identified to have functional importance in endothelial inflammatory responses but not in apoptosis. Furthermore, Zhou *et al*. demonstrated that oscillatory flow induces the expression of miR-21 at the transcriptional level in cultured ECs and eventually leads to an inflammatory response by targeting the 3′-UTR of the peroxisome proliferator-activated receptor-α (PPAR-α) 87. These studies provide a mechanism of atherogenic flow in which oscillatory flow induces an inflammatory response at the post-transcriptional level that is mediated by miRNAs.

Hastings *et al*. used an EC-VSMC co-culture model in response to atheroprone flow to identify the role of chromatin modifications in regulating the VSMC phenotypic switch 88. Mohamed *et al*. demonstrated that cyclic stretch-induced miR-26a serves as a hypertrophic gene; thus, the transcription factor CCAAT enhancer-binding protein directly activates miR-26a expression through the transcriptional machinery 89. In addition, miR-26a directly targets glycogen synthase kinase-3, an anti-hypertrophic protein, to enhance hypertrophy in VSMCs. In the animal study, Wu *et al*. found that miR-130a correlates with vascular remodelling in SHRs 90. MiR-130a was up-regulated in the thoracic aorta and mesenteric arteries of SHRs. In addition, the mRNA and protein levels of growth arrest-specific homeobox were down-regulated by miR-130a. MiR-130a mimics at 25 or 50 nmol/l significantly enhanced the proliferation of VSMCs. Yu *et al*. investigated the miRNA expression profile in isolated VSMCs from SHRs and WKYs 91. The let-7d miRNA was significantly down-regulated in VSMCs from SHRs, and the role of let-7d is supposedly related to the proliferation of VSMCs. In cellular assays, overexpression of let-7d directly targeted k-ras, which is an oncogene that participates in the modulation of the cell cycle and cell proliferation, leading to the inhibition of VSMC proliferation. This study implicates the role of let-7d in the mechanism of VSMC proliferation in hypertensive rats.

## Conclusions and future perspectives

Haemodynamic forces, such as fluid shear stress and cyclic stretch, can modulate EC and VSMC gene expression, cellular function and pathophysiology in health and disease. Although extensive studies have been performed on the molecular mechanisms by which haemodynamic forces regulate intracellular signals that ultimately modulate downstream gene expression, studies investigating the role of haemodynamic force-induced epigenetic pathways have emerged only recently. In this review, we summarize the current state of the *in vitro* and *in vivo* studies on haemodynamic force-induced DNA methylation, histone modification/remodelling and miRNA expression in the regulation of EC gene expression, cellular function and pathophysiology. Studies assessing eNOS gene expression, proliferation, angiogenesis, migration and vascular disorders, such as atherosclerosis and hypertension, are discussed. Shear stress-induced eNOS gene expression is regulated by epigenetic mechanisms, including the acetylation of histone H3 and H4, the interaction of p300 and NFκB with the eNOS promoter, and post-transcriptional modification by HDAC5 and miR-92a, which influences eNOS gene expression by directly binding to the 3′-UTR of KLF-2. These studies clearly demonstrate the complex regulation of eNOS gene expression by shear stress-induced epigenetic modification at the transcriptional and post-transcriptional level. HDACs are critical molecules that participate in multiple aspects of the EC response to shear stress. Particularly, shear stress-induced HDAC3 *via* the Flk-1–PI3K–Akt pathway controls EC differentiation, survival and proliferation. HDAC5 retards the disturbed flow-induced inflammatory response, leading to a decrease in adhesion molecules. In addition to HDACs, shear stress-modulated miRNAs, including miR126, miR-19a, miR-23b, miR-92a, miR-10a, miR-21 and miR-663, play a crucial role in EC angiogenesis, proliferation and atherosclerosis. The role of shear stress-induced epigenetic modifications in vascular physiology and pathophysiology is demonstrated by their regulatory roles in proliferation, angiogenesis, migration and atherosclerosis. In the SHR model, HDAC inhibitors, such as VPA and TSA, decrease blood pressure and hypertensive markers. The aberrant expression of let-7d, miR-130a and miR-26a may contribute to the VSMC proliferation observed during the development of hypertension.

Although extensive studies have revealed that shear stress-induced epigenetic modifications influence EC function, the role of other important mechanical forces, such as tensile forces from ECM remodelling, in the regulation of EC and VSMC functions remain unclear. During the development of atherosclerosis and hypertension, matrix remodelling is a critical feature as a growing number of studies have revealed that changes occur in matrix components and protease, such as MMPs alter the mechanical properties of vascular vessels 92. Haemodynamic force-induced epigenetic modifications are tightly correlated with physiological maintenance and pathophysiology. Similarly, mechanical forces derived from ECM remodelling may have functions in epigenetic modifications in vascular cells and should be investigated further. The study of epigenetic modifications will contribute to our understanding of the transcriptional and post-transcriptional control machinery in vascular disease that is stimulated by unusual mechanical forces, such as disturbed flow and oscillatory shear. Such studies will likely provide new insights into the mechanisms by which the dynamic environment of the vascular vessel influences the vascular cells during the development of vascular diseases.

## References

[1] Butcher DT, Alliston T, Weaver VM (2009). A tense situation: forcing tumour progression. Nat Rev Cancer.

[2] Chiu JJ, Chien S (2011). Effects of disturbed flow on vascular endothelium: pathophysiological basis and clinical perspectives. Physiol Rev.

[3] Jaalouk DE, Lammerding J (2009). Mechanotransduction gone awry. Nat Rev Mol Cell Biol.

[4] Hahn C, Schwartz MA (2009). Mechanotransduction in vascular physiology and atherogenesis. Nat Rev Mol Cell Biol.

[5] Van Speybroeck L (2002). From epigenesis to epigenetics: the case of C. H. Waddington. Ann NY Acad Sci.

[6] Goldberg AD, Allis CD, Bernstein E (2007). Epigenetics: a landscape takes shape. Cell.

[7] Portela A, Esteller M (2010). Epigenetic modifications and human disease. Nat Biotechnol.

[8] Moore LD, Le T, Fan G (2013). DNA methylation and its basic function. Neuropsychopharmacology.

[9] Kouzarides T (2007). Chromatin modifications and their function. Cell.

[10] Winter J, Jung S, Keller S (2009). Many roads to maturity: microRNA biogenesis pathways and their regulation. Nat Cell Biol.

[11] Chien S (2007). Mechanotransduction and endothelial cell homeostasis: the wisdom of the cell. Am J Physiol Heart Circ Physiol.

[12] Nguyen CT, Lu Q, Wang Y (2008). Zebrafish as a model for cardiovascular development and disease. Drug Discov Today Dis Models.

[13] Hove JR, Koster RW, Forouhar AS (2003). Intracardiac fluid forces are an essential epigenetic factor for embryonic cardiogenesis. Nature.

[14] Lucitti JL, Jones EA, Huang C (2007). Vascular remodeling of the mouse yolk sac requires hemodynamic force. Development.

[15] North TE, Goessling W, Peeters M (2009). Hematopoietic stem cell development is dependent on blood flow. Cell.

[16] Adamo L, Naveiras O, Wenzel PL (2009). Biomechanical forces promote embryonic haematopoiesis. Nature.

[17] Chiu JJ, Wang DL, Chien S (1998). Effects of disturbed flow on endothelial cells. J Biomech Eng.

[18] Berk BC, Abe JI, Min W (2001). Endothelial atheroprotective and anti-inflammatory mechanisms. Ann NY Acad Sci.

[19] Jo H, Sipos K, Go YM (1997). Differential effect of shear stress on extracellular signal-regulated kinase and N-terminal Jun kinase in endothelial cells. Gi2- and Gbeta/gamma-dependent signaling pathways. J Biol Chem.

[20] Li YS, Haga JH, Chien S (2005). Molecular basis of the effects of shear stress on vascular endothelial cells. J Biomech.

[21] Akimoto S, Mitsumata M, Sasaguri T (2000). Laminar shear stress inhibits vascular endothelial cell proliferation by inducing cyclin-dependent kinase inhibitor p21(Sdi1/Cip1/Waf1). Circ Res.

[22] Satcher R, Dewey CF, Hartwig JH (1997). Mechanical remodeling of the endothelial surface and actin cytoskeleton induced by fluid flow. Microcirculation.

[23] Matthews BD, Overby DR, Mannix R (2006). Cellular adaptation to mechanical stress: role of integrins, Rho, cytoskeletal tension and mechanosensitive ion channels. J Cell Sci.

[24] Li S, Chen BP, Azuma N (1999). Distinct roles for the small GTPases Cdc42 and Rho in endothelial responses to shear stress. J Clin Invest.

[25] Boon RA, Horrevoets AJ (2009). Key transcriptional regulators of the vasoprotective effects of shear stress. Hamostaseologie.

[26] Wang N, Miao H, Li YS (2006). Shear stress regulation of Kruppel-like factor 2 expression is flow pattern-specific. Biochem Biophys Res Commun.

[27] Li C, Xu Q (2000). Mechanical stress-initiated signal transductions in vascular smooth muscle cells. Cell Signal.

[28] Haga JH, Li YS, Chien S (2007). Molecular basis of the effects of mechanical stretch on vascular smooth muscle cells. J Biomech.

[29] Lemarie CA, Tharaux PL, Lehoux S (2010). Extracellular matrix alterations in hypertensive vascular remodeling. J Mol Cell Cardiol.

[30] Asanuma K, Magid R, Johnson C (2003). Uniaxial strain upregulates matrix-degrading enzymes produced by human vascular smooth muscle cells. Am J Physiol Heart Circ Physiol.

[31] Watase M, Awolesi MA, Ricotta J (1997). Effect of pressure on cultured smooth muscle cells. Life Sci.

[32] Birukov KG, Shirinsky VP, Stepanova OV (1995). Stretch affects phenotype and proliferation of vascular smooth muscle cells. Mol Cell Biochem.

[33] Li C, Wernig F, Leitges M (2003). Mechanical stress-activated PKCdelta regulates smooth muscle cell migration. FASEB J.

[34] Esteller M (2008). Epigenetics in evolution and disease. Lancet.

[35] Reik W, Dean W, Walter J (2001). Epigenetic reprogramming in mammalian development. Science.

[36] Deplus R, Brenner C, Burgers WA (2002). Dnmt3L is a transcriptional repressor that recruits histone deacetylase. Nucleic Acids Res.

[37] Rao X, Zhong J, Zhang S (2011). Loss of methyl-CpG-binding domain protein 2 enhances endothelial angiogenesis and protects mice against hind-limb ischemic injury. Circulation.

[38] Luger K, Mader AW, Richmond RK (1997). Crystal structure of the nucleosome core particle at 2.8 A resolution. Nature.

[39] Sterner DE, Berger SL (2000). Acetylation of histones and transcription-related factors. Microbiol Mol Biol Rev.

[40] Spin JM, Quertermous T, Tsao PS (2010). Chromatin remodeling pathways in smooth muscle cell differentiation, and evidence for an integral role for p300. PLoS ONE.

[41] Haberland M, Montgomery RL, Olson EN (2009). The many roles of histone deacetylases in development and physiology: implications for disease and therapy. Nat Rev Genet.

[42] Witt O, Deubzer HE, Milde T (2009). HDAC family: what are the cancer relevant targets?. Cancer Lett.

[43] Wang GG, Allis CD, Chi P (2007). Chromatin remodeling and cancer, Part I: covalent histone modifications. Trends Mol Med.

[44] Bolden JE, Peart MJ, Johnstone RW (2006). Anticancer activities of histone deacetylase inhibitors. Nat Rev Drug Discov.

[45] Xu WS, Parmigiani RB, Marks PA (2007). Histone deacetylase inhibitors: molecular mechanisms of action. Oncogene.

[46] Kim HJ, Bae SC (2011). Histone deacetylase inhibitors: molecular mechanisms of action and clinical trials as anti-cancer drugs. Am J Transl Res.

[47] Lusser A, Kadonaga JT (2003). Chromatin remodeling by ATP-dependent molecular machines. BioEssays.

[48] Mattick JS (2009). The genetic signatures of noncoding RNAs. PLoS Genet.

[49] Stefani G, Slack FJ (2008). Small non-coding RNAs in animal development. Nat Rev Mol Cell Biol.

[50] Erwin JA, Lee JT (2008). New twists in X-chromosome inactivation. Curr Opin Cell Biol.

[51] Small EM, Olson EN (2011). Pervasive roles of microRNAs in cardiovascular biology. Nature.

[52] Weber C, Schober A, Zernecke A (2010). MicroRNAs in arterial remodelling, inflammation and atherosclerosis. Curr Drug Targets.

[53] Denli AM, Tops BB, Plasterk RH (2004). Processing of primary microRNAs by the Microprocessor complex. Nature.

[54] Chendrimada TP, Gregory RI, Kumaraswamy E (2005). TRBP recruits the Dicer complex to Ago2 for microRNA processing and gene silencing. Nature.

[55] Gregory RI, Chendrimada TP, Cooch N (2005). Human RISC couples microRNA biogenesis and posttranscriptional gene silencing. Cell.

[56] Fish JE, Santoro MM, Morton SU (2008). miR-126 regulates angiogenic signaling and vascular integrity. Dev Cell.

[57] Harris TA, Yamakuchi M, Ferlito M (2008). MicroRNA-126 regulates endothelial expression of vascular cell adhesion molecule 1. Proc Natl Acad Sci USA.

[58] Cordes KR, Sheehy NT, White MP (2009). miR-145 and miR-143 regulate smooth muscle cell fate and plasticity. Nature.

[59] Tai SC, Robb GB, Marsden PA (2004). Endothelial nitric oxide synthase: a new paradigm for gene regulation in the injured blood vessel. Arterioscler Thromb Vasc Biol.

[60] Illi B, Nanni S, Scopece A (2003). Shear stress-mediated chromatin remodeling provides molecular basis for flow-dependent regulation of gene expression. Circ Res.

[61] Fish JE, Matouk CC, Rachlis A (2005). The expression of endothelial nitric-oxide synthase is controlled by a cell-specific histone code. J Biol Chem.

[62] Lund G, Andersson L, Lauria M (2004). DNA methylation polymorphisms precede any histological sign of atherosclerosis in mice lacking apolipoprotein E. J Biol Chem.

[63] Post WS, Goldschmidt-Clermont PJ, Wilhide CC (1999). Methylation of the estrogen receptor gene is associated with aging and atherosclerosis in the cardiovascular system. Cardiovasc Res.

[64] Kim J, Kim JY, Song KS (2007). Epigenetic changes in estrogen receptor beta gene in atherosclerotic cardiovascular tissues and in-vitro vascular senescence. Biochim Biophys Acta.

[65] Zeng L, Zhang Y, Chien S (2003). The role of p53 deacetylation in p21Waf1 regulation by laminar flow. J Biol Chem.

[66] Lee DY, Lee CI, Lin TE (2012). Role of histone deacetylases in transcription factor regulation and cell cycle modulation in endothelial cells in response to disturbed flow. Proc Natl Acad Sci USA.

[67] Zampetaki A, Zeng L, Margariti A (2010). Histone deacetylase 3 is critical in endothelial survival and atherosclerosis development in response to disturbed flow. Circulation.

[68] Zeng L, Xiao Q, Margariti A (2006). HDAC3 is crucial in shear- and VEGF-induced stem cell differentiation toward endothelial cells. J Cell Biol.

[69] Chen W, Bacanamwo M, Harrison DG (2008). Activation of p300 histone acetyltransferase activity is an early endothelial response to laminar shear stress and is essential for stimulation of endothelial nitric-oxide synthase mRNA transcription. J Biol Chem.

[70] Huddleson JP, Ahmad N, Srinivasan S (2005). Induction of KLF2 by fluid shear stress requires a novel promoter element activated by a phosphatidylinositol 3-kinase-dependent chromatin-remodeling pathway. J Biol Chem.

[71] Illi B, Dello RussoC, Colussi C (2008). Nitric oxide modulates chromatin folding in human endothelial cells via protein phosphatase 2A activation and class II histone deacetylases nuclear shuttling. Circ Res.

[72] Wang W, Ha CH, Jhun BS (2010). Fluid shear stress stimulates phosphorylation-dependent nuclear export of HDAC5 and mediates expression of KLF2 and eNOS. Blood.

[73] Wang YH, Yan ZQ, Qi YX (2010). Normal shear stress and vascular smooth muscle cells modulate migration of endothelial cells through histone deacetylase 6 activation and tubulin acetylation. Ann Biomed Eng.

[74] Yan ZQ, Yao QP, Zhang ML (2009). Histone deacetylases modulate vascular smooth muscle cell migration induced by cyclic mechanical strain. J Biomech.

[75] Chen Z, Peng IC, Cui X (2010). Shear stress, SIRT1, and vascular homeostasis. Proc Natl Acad Sci USA.

[76] Stein S, Matter CM (2011). Protective roles of SIRT1 in atherosclerosis. Cell Cycle.

[77] Cardinale JP, Sriramula S, Pariaut R (2010). HDAC inhibition attenuates inflammatory, hypertrophic, and hypertensive responses in spontaneously hypertensive rats. Hypertension.

[78] Bogaard HJ, Mizuno S, Hussaini AA (2011). Suppression of histone deacetylases worsens right ventricular dysfunction after pulmonary artery banding in rats. Am J Respir Crit Care Med.

[79] Usui T, Okada M, Hara Y (2011). Exploring calmodulin-related proteins, which mediate development of hypertension, in vascular tissues of spontaneous hypertensive rats. Biochem Biophys Res Commun.

[80] Balligand JL, Feron O, Dessy C (2009). eNOS activation by physical forces: from short-term regulation of contraction to chronic remodeling of cardiovascular tissues. Physiol Rev.

[81] Wu W, Xiao H, Laguna-Fernandez A (2011). Flow-dependent regulation of Kruppel-Like factor 2 Is mediated by microRNA-92a. Circulation.

[82] Nicoli S, Standley C, Walker P (2010). MicroRNA-mediated integration of haemodynamics and Vegf signalling during angiogenesis. Nature.

[83] Qin X, Wang X, Wang Y (2010). MicroRNA-19a mediates the suppressive effect of laminar flow on cyclin D1 expression in human umbilical vein endothelial cells. Proc Natl Acad Sci USA.

[84] Wang KC, Garmire LX, Young A (2010). Role of microRNA-23b in flow-regulation of Rb phosphorylation and endothelial cell growth. Proc Natl Acad Sci USA.

[85] Fang Y, Shi C, Manduchi E (2010). MicroRNA-10a regulation of proinflammatory phenotype in athero-susceptible endothelium in vivo and in vitro. Proc Natl Acad Sci USA.

[86] Ni CW, Qiu H, Jo H (2011). MicroRNA-663 upregulated by oscillatory shear stress plays a role in inflammatory response of endothelial cells. Am J Physiol Heart Circ Physiol.

[87] Zhou J, Wang KC, Wu W (2011). MicroRNA-21 targets peroxisome proliferators-activated receptor-alpha in an autoregulatory loop to modulate flow-induced endothelial inflammation. Proc Natl Acad Sci USA.

[88] Hastings NE, Simmers MB, McDonald OG (2007). Atherosclerosis-prone hemodynamics differentially regulates endothelial and smooth muscle cell phenotypes and promotes pro-inflammatory priming. Am J Physiol Cell Physiol.

[89] Mohamed JS, Lopez MA, Boriek AM (2010). Mechanical stretch up-regulates microRNA-26a and induces human airway smooth muscle hypertrophy by suppressing glycogen synthase kinase-3beta. J Biol Chem.

[90] Wu WH, Hu CP, Chen XP (2011). MicroRNA-130a mediates proliferation of vascular smooth muscle cells in hypertension. Am J Hypertens.

[91] Yu ML, Wang JF, Wang GK (2011). Vascular smooth muscle cell proliferation is influenced by let-7d microRNA and its interaction with KRAS. Circ J.

[92] Raines EW (2000). The extracellular matrix can regulate vascular cell migration, proliferation, and survival: relationships to vascular disease. Int J Exp Pathol.

